# Scanning ion conductance microscopy of live human glomerulus

**DOI:** 10.1111/jcmm.16475

**Published:** 2021-03-21

**Authors:** Ruslan Bohovyk, Mykhailo Fedoriuk, Elena Isaeva, Andrew Shevchuk, Oleg Palygin, Alexander Staruschenko

**Affiliations:** ^1^ Department of Physiology Medical College of Wisconsin Milwaukee WI USA; ^2^ Department of Cellular Membranology Bogomoletz Institute of Physiology Kiev Ukraine; ^3^ Department of Medicine Imperial College London London UK; ^4^ Clement J. Zablocki VA Medical Center Milwaukee WI USA

## Abstract

Podocyte damage is a hallmark of glomerular diseases, such as focal segmental glomerulosclerosis, typically associated with marked albuminuria and progression of renal pathology. Podocyte structural abnormalities and loss are also linked to minimal change disease and more common diabetic kidney disease. Here we applied the first‐time scanning ion conductance microscopy (SICM) technique to assess the freshly isolated human glomerulus's topology. SICM provides a unique opportunity to evaluate glomerulus podocytes as well as other nephron structural segments with electron microscopy resolution but in live samples. Shown here is the application of the SICM method in the live human glomerulus, which provides proof of principle for future dynamic analysis of membrane morphology and various functional parameters in living cells.

## 1 INTRODUCTION

Glomerulus represents a tuft of capillaries located at the entry portion of the nephron, where blood is selectively filtered across the glomerular filtration barrier consisting of endothelial cells, glomerular basement membrane, and podocytes. Primary and secondary processes of adjacent podocytes intertwine, creating a slit diaphragm that tightly covers glomeruli capillaries. This diaphragm has a complex morphology and forms the final barrier for blood filtration contributing to size selectivity and permitting permeability to molecules smaller than albumin. A reduction in podocyte number and foot process effacement was reported in focal segmental glomerulosclerosis, minimal change disease and diabetic nephropathy.^1^, ^2^, ^3^ Moreover, podocytes’ loss and modification of their architecture can be evident at the very early stages of kidney diseases that may underlie/exacerbate their progression. High‐resolution imaging of cell morphology is becoming an essential and useful tool for studying cellular structures and the role they play in cell function and disease progression.^4^ Scanning electron microscopy (SEM) is a widely used method for conducting such studies. Still, it requires multiple complex procedures for sample preparation, making it impossible to analyse a living sample.^5^ Therefore, there is a critical need to develop new tools to study alterations of podocyte morphology in live samples. Hopping Probe mode of Scanning Ion Conductance Microscopy (SICM) is a technique that enables high‐resolution, non‐optical imaging of living cell surfaces with complex morphology.^6^, ^7^, ^8^ The major advantage of this method is a close to several nanometres spatial resolution and a long Z (vertical) range, which can be applied to live samples with convoluted morphology under physiologically relevant conditions. SICM is a multimodal imaging technique that can be combined with other established techniques, concurrent and dynamic analysis of membrane morphology and various functional parameters, such as cell volume, membrane potentials, single ion‐channel currents and even the dynamics of membrane protein complexes in living cells.^9^, ^10^, ^11^ Here, we provide an example of the application of SICM to analyse the morphology of live human glomerulus structure.

## 2 MATERIALS AND METHODS

**FIGURE 1:**
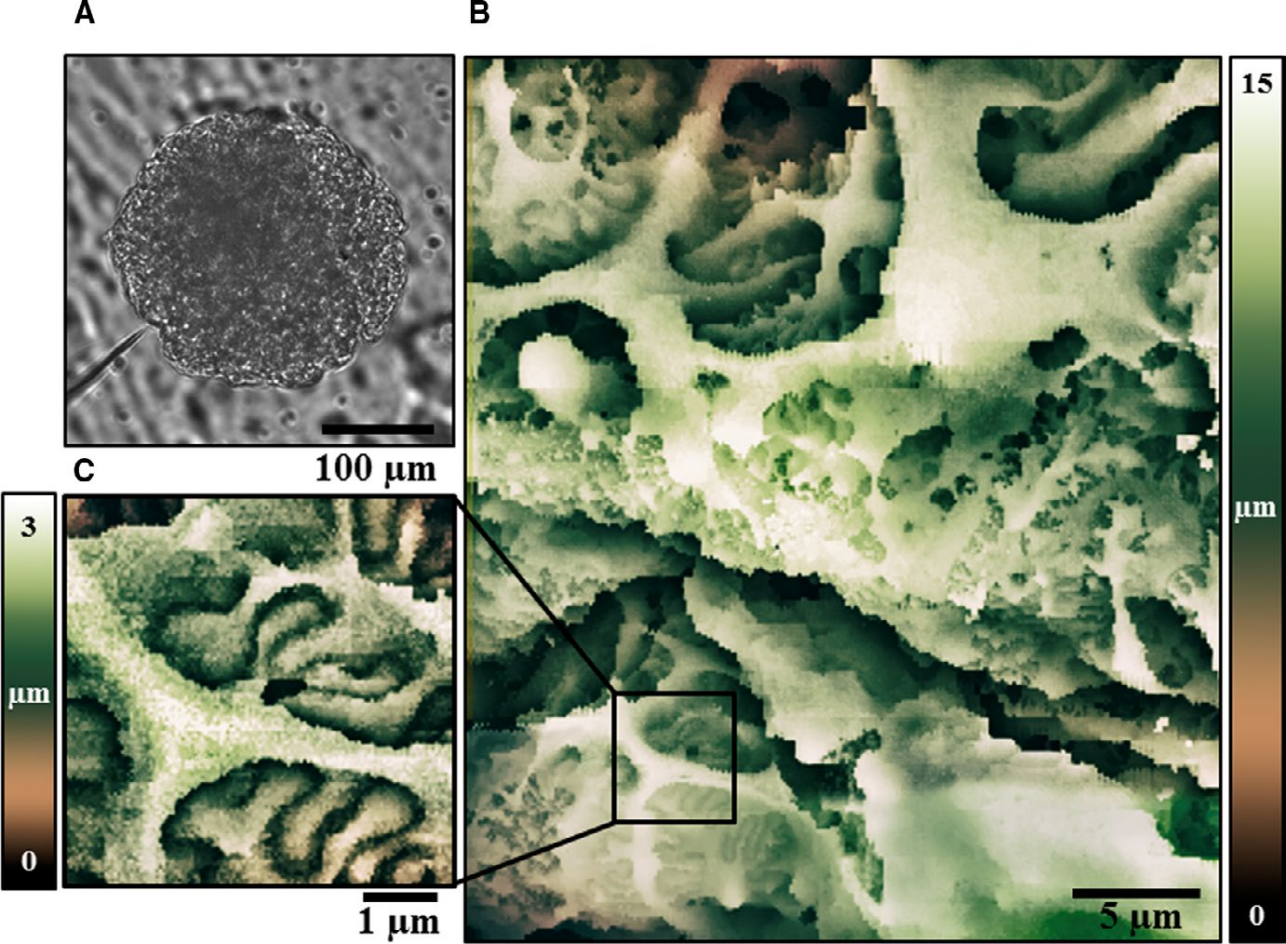
Application of Scanning Ion Conductance Microscopy imaging. A, Freshly isolated human glomerulus attached to poly‐L‐lysine glass surface. Shown on the left is a micropipette approaching the glomerulus surface to perform SICM imaging. B, An example of glomerulus filtration barrier components, including podocyte and foot processes covered vessels, visualized by SICM. 45 min for total area scan (30 × 30 µm, resolution 512 × 512 pixels; z‐axis changes 15 µm). C, Expended view on the architecture of human podocyte secondary foot processes revealed by high‐resolution SICM. 12 min for total area scan (resolution 5 × 5 µm, resolution 480 × 480 pixels; z‐axis changes 3 µm). Scale bars are shown

For the experiment, we used a part of the cortical area from the discarded transplant human kidney dissected and stored in Wisconsin preservation solution followed by the incubation in an oxygenated physiological saline solution (PSS).^12^ Renal glomeruli were isolated using a vibrodissociation technique as previously described.^13^ This approach allows the rapid isolation of well‐preserved renal glomeruli from human kidneys. To perform SICM imaging, freshly isolated human glomerulus were attached to poly‐L‐lysine glass surface placed into the cell chamber filled with PSS solution. Samples were manually positioned in the x‐y directions under an inverted optical microscope Nikon TE2000‐U (Nikon Instruments). For the hopping probe, SICM imaging borosilicate glass nanopipettes with a resistance of approximately 100 MΩ, which corresponds to an estimated tip diameter around 120 nm, were used. Nanopipettes were pulled with the horizontal flaming/brown type puller P‐97 (Sutter Instruments, Novato, CA). The nanopipettes were filled with the same PSS solution used for the bath and were positioned in the z‐direction with a piezoelectric actuator. The ion current flowing through the nanopipettes was measured with an Axopatch 700B patch‐clamp amplifier (Axon Instruments) in voltage‐clamp mode and monitored by the custom‐modified universal controller (ICAPPIC Ltd, UK), which simultaneously controlled sample and pipette positioning.^6^ All experiments were conducted at room temperature (20‐22°C). For imaging complex convoluted structures such as podocytes foot processes in the glomerulus, we used backstep/hopping mode SICM that is most applicable for imaging the topography of living cells with high resolution.^6^, ^14^ The single step of the hopping principle consists of an event when the reference current is measured by positioning the nanopipette well above the sample. Then, the nanopipette approaches along the z‐axis to the sample surface until the measured current level drops to the preset set‐point corresponding to ~ 1.0%‐1.2% of the reference current. At this moment, the approach is terminated, the xyz‐coordinates of the XY and Z piezo actuators are recorded as the sample surface coordinate for the first imaging point, and the pipette is withdrawn away from the surface. This procedure then repeats at each single imaging point along x‐y axis determined by preset resolution and scan area range. For Figure 1B, scan range and image resolutions were 30 µm × 30 µm and 512 px × 512 px, and for Figure 1C were 5 µm × 5 µm and 480 px × 480 px, respectively. Raw SICM topography data and image post‐processing were performed using SICM ImageViewer microscopy analysis software (ICAPPIC Ltd, UK).

## 3 RESULTS AND DISCUSSION

SICM image quality is comparable to SEM and allows precise examination of glomerulus surface structure, including blood vessels and podocytes, and the architecture of the secondary foot processes.^4^ In our previous study, we used SICM imaging to estimate the pathological structural changes in podocyte foot processes in type 2 diabetic nephropathy (T2DN) rats.^15^ We conducted a comparative analysis of the three‐dimensional architecture of the podocyte filtration barrier in non‐diabetic and T2DN rats. Our data revealed a significant loss of podocyte foot processes in T2DN glomeruli providing an accurate evaluation for histopathological changes occurred in diabetic nephropathy that underlie nephrinuria and albuminuria in these rats.^15^ Here, for the first time, we demonstrated a successful approach in assessing the three‐dimensional surface structure of freshly isolated, live human glomerulus. Human renal glomeruli are spherical structures with a diameter of around 300 µm^16^; thus, the tip of the pipette must carefully approach the top of the region of interest. Our SICM set‐up has 25 µm z‐axis and 30 × 30 µm x‐y‐axis scanner range, which allowed us to obtain a high‐resolution image of the large part of the glomerulus surface. The acquisition time strongly depends on the scan range, resolution and hopping electrode tip size. For live‐cell imaging in complex structures like freshly isolated glomerulus, the scanning time varies from 10 to 50 minutes per frame (see Figure legend for more details). The cumulative effect of the high resolution, large scanning area and stochastic fluctuations in living systems may result in imaging artefacts represented as horizontal and vertical shifts between hoping pipette reposition cycles. Note that in complex geometries with the sudden changes in the surface locations, both the acquisition time and imaging artefacts, like dark or bad pixels, could increase. The SICM topography image (Figure 1A) illustrates glomerular capillaries covered by clearly visible primary and secondary podocyte foot processes. SICM allows acquiring sample images with different scan ranges in the nanometre scale, keeping a sufficient resolution level.^17^ Figure 1B represents the architecture of human podocyte secondary foot processes revealed by high‐resolution SICM. Further analysis of such topography images can be used to study morphological changes of podocyte foot processes under pathological conditions and in response to various drug applications.

The application of the SICM technique for investigating the three‐dimensional surface structure of the soft tissue samples, including glomerulus and podocyte cells, was initially introduced by Nakajima et al^4^. The authors used kidney slices to compare this technique with conventional SEM. Here, we extended these studies to live human glomerulus. Using this approach, we revealed the great potential of the SICM technique for the quick and reliable examination of the glomerulus filtration barrier. One of the great advantages of SICM (in addition to that the use of live samples) is that it does not require multiple sample preparation procedures typically needed for EM microscopy imaging, including dehydration and fixation solution. Therefore, SICM allows avoiding shrinkage of samples observed with EM, which may lead to misleading of actual dimensions. Importantly, real‐time SICM on freshly isolated glomeruli also allows testing the dynamic of foot processes movement in response to the acute exposure to drugs of interest. Finally, the SICM pipette can be used to perform patch‐clamp recordings of single‐channel currents.^18^ For this purpose, after the reconstruction of the topography map, the acquisition software (ICAPPIC Ltd, UK) allowed for a controlled brake of the SICM glass hopping probe on the chamber surface, decreasing microelectrode resistance to the patch‐clamp conditions (7 to 12 MΩ) and making it suitable for the single‐channel recording in the same experiment. The SICM permits the selection of the patch‐clamp electrode's exact location on the cell surface based on topographical data by a well‐controlled vertical approach with nanometre precision, resulting in the easy formation of a contact gigaseal with the cellular membrane called Smart Patch.^19^

In summary, the method described here has excellent potential for the studies of glomeruli and other freshly isolated nephron segments. Furthermore, it shows that it is possible to use human kidney samples without elaborate preparation to evaluate morphological changes in the study of pathologies.

## CONFLICT OF INTEREST

Dr Andrew Shevchuk is a shareholder and receives the consulting fees from the ICAPPIC Ltd. All other authors declared no competing interests.

## AUTHOR CONTRIBUTIONS

**Ruslan Bohovyk:** Conceptualization (equal); Data curation (lead); Formal analysis (lead); Writing‐original draft (equal); Writing‐review & editing (equal). **Mykhailo Fedoriuk:** Data curation (supporting); Formal analysis (supporting); Writing‐review & editing (equal). **Elena Isaeva:** Conceptualization (supporting); Data curation (supporting); Formal analysis (supporting); Writing‐original draft (supporting); Writing‐review & editing (equal). **Andrew Shevchuk:** Conceptualization (supporting); Software (supporting); Writing‐original draft (supporting); Writing‐review & editing (equal). **Oleg Palygin:** Conceptualization (equal); Formal analysis (supporting); Methodology (supporting); Project administration (supporting); Writing‐original draft (supporting); Writing‐review & editing (equal). **Alexander Staruschenko:** Conceptualization (lead); Project administration (lead); Resources (lead); Supervision (lead); Writing‐original draft (equal); Writing‐review & editing (equal).

## DATA AVAILABILITY STATEMENT

The data that support the findings of this study are available from the corresponding author upon reasonable request.
